# The psychosocial impact of home use medical devices on the lives of older people: a qualitative study

**DOI:** 10.1186/1472-6963-13-467

**Published:** 2013-11-06

**Authors:** Ross Thomson, Jennifer L Martin, Sarah Sharples

**Affiliations:** 1 Faculty of Engineering, University of Nottingham, Tower Building, University Park Nottingham, Nottingham NG7 2RD, UK; 2 Faculty of Engineering, A44 Coates Building, University Park, Nottingham NG7 2RD, UK

**Keywords:** Home environment, Medical devices, Older people, Psychosocial issues, Qualitative research

## Abstract

**Background:**

Increased life expectancy and the accompanying prevalence of chronic conditions have led to the focus and delivery of health care migrating from the hospital and into people’s homes. While previous studies have investigated the integration of particular types of medical devices into the home, it was our intention to describe how medical devices are integrated into the lives of older people.

**Methods:**

Adopting a qualitative study design, 12 older people, who used medical devices in the home, took part in in-depth, semi structured interviews. In 7 of the interviews participants and their partners were interviewed together. These interviews were recorded, transcribed and analysed thematically.

**Results:**

Two themes were constructed that describe how medical devices that are used in the home present certain challenges to older people and their partners in how the device is adopted and the personal adaptations that they are required to make. The first theme of 'self-esteem’ highlighted the psychological impact on users. The second theme of 'the social device' illustrated the social impact of these devices on the user and the people around them.

**Conclusions:**

We found that these devices had both a positive and negative psychosocial impact on users’ lives. An improved understanding of these psychological and social issues may assist both designers of medical devices and the professionals who issue them to better facilitate the integration of medical devices into the homes and lives of older people.

## Background

The reported increased life expectancy that is being seen in Western Europe, Japan and the United States of America is mirrored by a rise in chronic diseases [1]. These chronic diseases are increasingly being controlled and monitored by patients themselves, thus facilitating the transfer of the health care setting for older people away from the hospital and into communities and homes. This relocation of health care has resulted in the increased use of medical devices in the home [2,3].

Studies that have looked at the user experience of different types of medical devices in the home have shown that users are required to make adjustments to the way that they live in their own homes. In a study by Ingadottir and Jonsdottir [4], oxygen compressors were considered to be large and noisy objects that have to be kept outside the bedroom when used at night because of the noise. The spouse of one participant took to sleeping in a separate room due lack of sleep as a result. In another study, a participant who used peritoneal dialysis complained that the bedroom now resembled a hospital room, while users receiving parenteral nutrition or oxygen therapy were concerned about the visual impact of the treatment equipment and would either attempt to hide the equipment from visitors or invite people to the home less often [5]. On the whole, home dialysis and oxygen therapy equipment seems to be experienced as bulky and conspicuous, having a detrimental effect on the aesthetics of the home environment. Efforts are made to conceal this equipment in a bid to reduce the institutionalised look of the home [6].

The home environment is particularly important to the elderly with growing old being associated with an increased and intensified attachment and emotional involvement in familiar places [7]. It has been suggested that clinical interventions might have a transformative effect on the inherent characteristics of the home and create ambiguities between 'home’ and 'institution’ which may erase or reverse any positive notions that older people might have for the home [8,9]. It is also worth noting that older people might have spouses and family members living with them, all of whom will also have an attachment to the home. Thus, the presence of medical equipment and the possibility of having to interact with the devices or help with treatment may have a bearing on their own roles and identity within that environment [10].

The literature around home use medical devices has identified areas of tension between users in general and specific medical devices. In our study we collected and analysed qualitative data in an attempt to describe how medical devices are integrated into the lives of older people.

## Methods

### Participants

Following approval by the University of Nottingham Faculty of Engineering Ethical Review Committee a sample of 12 participants and 7 of their partners was recruited from the East Midlands. Participants and their partners were interviewed together. The inclusion criteria were that participants had to be 65 years or older. The age of the participants ranged from 65 to 83 years (mean 72 years) and 6 were female. The age of the 7 partners ranged from 65 to 82 years (mean 72) and 4 were female. Participants also had to be currently using a medical device in the home. The definition of a medical device was taken from the Medicines and Health care products Regulatory Agency (MHRA) which is an executive agency of the Department of Health. The MHRA describes the term 'medical device’ as covering “all products, except medicines, used in health care for the diagnosis, prevention, monitoring or treatment of illness or disability” [11]. This definition includes some products that may traditionally be thought of as assistive devices (Table 1).

**Table 1:** Participant and device details

**Participant number**	**Devices and duration of use**	**Partner present**
1	Transcutaneous electrical nerve stimulation device > 1 year	Yes
	Leg circulation booster < 1 year	
2	Blood pressure monitor >5 years	Yes
	Blood glucose meter >5 years	
3	Oxygen concentrator 8 months	Yes
	Portable oxygen tank 1 year	
	Nebulizer >1 year	
	Stairlift >2 years	
4	Continuous ambulatory peritoneal dialysis 4 years	Yes
	Automatic peritoneal dialysis 8 months	
5	Blood pressure monitor 6 years	No
6	Implantable cardioverter-defibrillator monitor 6 months	Yes
7	Transcutaneous electrical nerve stimulation device 3 months	No
	Telecare monitor >10 years	
8	Stair-lift >3 years	Yes
9	Nebulizer >3 years	No
10	Nebulizer >5 years	Yes
	Blood pressure monitor >5 years	
	Sats monitor >3 years	
	Blood glucose meter >3 years	
11	Blood pressure monitor 8 years	No
12	Blood pressure monitor 1 year	No
	Mobility device 2 years	

To capture the idea of 'home’ in home use medical devices and limit some of the variability inherent within the term, participants were excluded if they lived in nursing/care homes as it was thought that medical devices would be more readily accepted in these types of establishments.

Participants were recruited via newspaper ads, posters placed in shop windows and emails sent to different support and older people’s groups. Due to low response rates from these recruitment methods participants were also asked to introduce the study to other potential participants within their social network, a practice known as snowball sampling. All participants were fully informed about the study and consent forms were completed prior to the interviews.

### Data collection

Semi-structured interviews were guided by an interview schedule consisting of 11 open-ended questions, some of which, because of their broad exploratory nature, were followed up with more focused prompts if required. The interview began with questions regarding the devices themselves (e.g. usage and storage). This was followed by questions about the participant’s home. The final part of the interview encouraged participants to consider how they felt the device fitted into their lives. Interviews lasted approximately 60 minutes and upon completion interviewees were given a full de-brief with contact numbers for places of further support. The first author (RT) carried out the interviews which were recorded digitally and subsequently transcribed verbatim. RT also analysed and interpreted the data. The interviews were conducted either at the participant’s home or at a public place according to the wishes of the participants. Participants and their partners were interviewed together.

### Analysis

Braun and Clarke’s [12] systematic approach to thematic analysis was used to identify, analyse and report the themes within the interview data. According to their method, thematic analysis can be divided into 6 separate stages. Stage 1 starts with engaging and familiarising oneself with the data. Stage 2 involves the systematic generation of initial codes, “the most basic segment, or element, of the raw data or information that can be assessed in a meaningful way regarding the phenomenon” [13] (p63). In stage 3 codes are then analysed and combined to produce themes that best “capture the essence of the phenomenon under investigation” [14] (p258). Once a preliminary set of themes is produced stage 4 is to review those themes ensuring that they related back to the data. Further theme analysis, stage 5, is required to generate clear definitions and relevant theme names before the final stage 6 which entails producing the final report.

Transcripts were analysed adopting a critical realist perspective [15], recognizing that it is possible to acquire an insight into older people’s experiences of using medical devices in the home through their accounts, but also that as researchers we have a role in constructing that knowledge. Thus the use of critical realism allowed the interview data to be collected and analysed in terms of what was being said as one possible way of trying to understand the underlying mechanisms and generative causations embedded within social structures [16].

As proposed by Creswell [17] an independent audit was carried out. This process involved a researcher, who was not part of the study, examining both the process and narrative account in order to verify the trustworthiness and credibility of the findings.

## Results

Two major themes were identified in the analysis which describes how medical devices are integrated into the lives of older people and are presented in the following sections: 'striving to maintain self-esteem’ and 'the social device’.

Quotes from device users and their partners/spouse will be identified in the following manner; participant 1 (P1), partner/spouse of participant 1 (PP1).

### Striving to maintain self-esteem

The medical devices in this study were not without consequences for participants’ self-esteem and seemed to include a range of different feelings and techniques that were organised into the subthemes of: feeling powerless, experiencing personal control, mastering the device and comparing oneself to others.

### Feeling powerless

Some participants considered that using medical devices was just something you had to do and were very conscious of the potentially fatal consequences of not doing so. There was a sense of resignation and powerlessness surrounding their illness and device use that would have had a detrimental effect on self-esteem.

*'It was just something we had to do. Now, it's just what I've got to do and the alternative was… Death, really. . . There is no alternative so you take what comes at you. . . . it doesn’t make you very happy but that's your fate and I've got you know, kismet’* (P4).

*'[B]ut I don't necessarily like doing it, but it's gotta be done If you don't do it, you won't be doing it. . . . You realize you've, it's a fact of life, you've gotta do it’* (P9).

The following example highlights how it is not just the device user who can be left feeling powerless and that the change in situation is also forced upon the partner.

*'No. We had no alternative anyway. . . What do you think? I think we’ve adapted don’t you?* (PP10)

*'Well I think we’ve had to adapt’* (P10).

*[It was] a big change for us so I think we’ve adapted to them and we just get on with it because we know we have to. . . We don’t really have a choice do we? Because if [he] didn’t have all these things, he probably wouldn’t be around’* (PP10).

### Experiencing personal control

Participants reported the importance of using devices to monitor their condition and how this enhanced feelings of control over their illness.

*'Well, I use my [glucose] monitor seven or eight times a day probably. . . . That’s so I can just, I always know what state my blood sugar’s in’* (P2).

*'[with my blood pressure monitor] I feel in control of managing the high blood pressure I think, and I know what it is. . . . You’re not in control if you don’t have some way of measuring it yourself’* (P11).

*'And my blood pressure thing it helps because if I find one day that it’s up a bit then I will curtail what I’m doing and relax and rest and change what I’m doing that day to adapt myself so my blood pressure goes down again’* (P12).

This was not just limited to devices that are designed to monitor or measure medical conditions. Participants using devices to deliver therapies or drugs also reported ways in which their device enabled them to exert personal control over their illness.

*Well, it is a tie [dialysis]. It's something you've got to remember to do, you can hardly forget but I learnt that you can push dialysis together . . . so you can have one or two quickens and then stretch one out and things like that, when I realised that that made life easier’* (P4).

### Mastering the device

Mastering the device was also important in bolstering self-esteem and its positive effect can be heard in the sense of pride expressed by some of the interviewees.

*'You’ve got to know how to use the equipment. Luckily I’ve been shown properly and I’ve always used it in the correct manner and it’s always worked’* (P10).

*And then when I first sat on [the stairlift], I always remember the first night . . . I, sort of, got a bit anxious about it first of all. [people said] “You’ll be alright. Just keep your finger on that,” you know, on the little switch thing, because directly you take it off, of course, it stops.And then now, I think to myself, “Oh, I wish the thing would go faster,” you know, but when I first had it I thought, “Oh, this is just right,” you know, “and it’s nice and slow and everything,” but now you think, “Oh, it’s very slow. I wish it would go faster.” Because they don’t go very fast’* (P8).

*'[It is important to] Make sure you do it properly. Make sure you are relaxed when you do it and make sure you know exactly what you are doing . . . so make sure that you’ve read the little booklet that goes with it so you know exactly what they mean and what they don’t mean’* (P12).

Again this is not dependent on whether the device is designed to monitor or to deliver therapy and participant 12 reported instances in which she used both personal control and mastery to reduce feelings of powerlessness.

### Comparing oneself to others

Some participants bolstered their self-esteem by comparing themselves to other device users who were not as in control of their situation or did not use the device correctly.

*'There are people that have got [telecare emergency buttons] here and they leave them on the sideboard or the kitchen cabinets, they forget to put them on and [the carers] always say to me it’s lovely to come and see you and that is round your neck. I just say to them look darling that could save my life’* (P7).

*'Well I’ve seen people using nebulisers who haven’t a clue what to do and not actually breathing the air in properly because they’ve not been actually been told how to use the mask and various equipment.* (P10) . . . *we always wash his nebuliser every time he uses it, every single time and we know people who don’t’* (PP10).

The following examples illustrate how some of these comparisons allowed participants to derogate or belittle others in a similar situation to themselves in order to maintain or bolster self-esteem.

*'And it’s amazing the number of people I’ve met who know absolutely nothing about [diabetes] and they’re sitting there and we got talking about it and they know virtually nothing about what’s what, the situation they’re in . . .’* (P2).

*'It’s true. People get equipment and are given things and they don’t do what they are told to do . . . Sometimes [my brother-in-law] misses pills or it’s “Oh I forgot to do this… or I couldn’t be bothered doing that”. Well you’re never going to get the best out of anything unless you do what you’re supposed to do’* (P10).

### The social device

Home use medical devices are not used in isolation and as such participants reported different ways in which the medical devices they used influenced their social interactions. These allowed the medical devices to be thought as either 'disrupting social harmony’ or 'bringing people together’.

### Disrupting social harmony

Some participants reported that device use had a negative impact on their partners. For example the actual process of using the device became problematic and meant having to organise when the device was used in order to maintain a social harmony:

*'At first it was a little bit of a problem between my husband and I because he kept on saying, “Why do you keep on taking your blood pressure?. . . You don’t have to keep taking [it] …” so I now do it usually when he’s not around. I don’t know why he gets uppity about it, but it seems to make him feel a bit annoyed that I keep doing it’* (P12).

Another participant who used a device overnight explained some of the problems with the device itself and how that affected sleeping arrangements because of alarms going off every time he turned over in bed:

*The problems [my wife] had I think [were] more than me at night with a buzzer going off . . .* (P4). *'If I got to the point where I was exhausted, I would have, yes, I would have slept in a separate bedroom on occasion to get an undisturbed night. . . . And we had to change sides because of the way the bedroom is laid out and the best way to do it was to have it on what is my side. So we had to change sides. That was strange!* (PP4).

Devices did not just present a barrier between couples but also caused concerns within a wider social context. Using their device in front of others was deemed to be problematic for some:

*“[using the device in public] . . . I could see the anxiety on their faces. Is he all right? . . . So I stopped doing it. Went and hid myself and did it on my own.”* (P9).

One couple reported the embarrassment felt by them both due to the noise produced by a portable oxygen cylinder and a conserver device when out shopping:

*'The only problem to having the portable . . .’* (P3).

*'We felt a bit embarrassed when you were shopping’* (PP3).

*'Yes, I was going to say that. That’s the only bit that I didn’t like about it was when I’d got to go out shopping with the oxygen. I just felt embarrassed, which obviously, I know I shouldn’t do but yes, I do feel that’* (P3,).

Interestingly in the last quote the partner says that '*we* felt a bit embarrassed’. It is unclear if he meant that he felt embarrassed or whether it is an example of how medical devices can bring people together as in the next subtheme.

### Bringing people together

Not all medical device use in the home environment presented social barriers. There was evidence to suggest that devices were also instrumental in bringing people together. One participant commented on how she considered the device was responsible for bringing herself and her daughter closer together:

*'So she’s realised mum isn’t quite so invincible and mum is going to go one day and what have you so it makes her … in a way it makes her love me a bit more’* (P12).

It also seems that both the user and partner had adopted joint ownership and responsibility for the devices in that when partners were talking they used the pronoun 'we’ or 'us’:

*'And****we’ve****got a standby cylinder for the inhaler … for the concentrator, if there’s a power failure’* (PP3).

*'Oh yeah. That’s what they did before when they gave****us****a new one [nebuliser]. Something went wrong and they didn’t know what it was but they gave****us****a new one straight away, they’re very good’* (PP10).

The idea of joint ownership and that a medical device can be more than a personal item limited to one designated user was expressed in other ways. It was not uncommon to find that people were willing to share their device with others and that the devices themselves became accepted and part of family life. For instance devices were shared with family members who suffered similar problems:

*'. . . like my son-in-law had when he had a bad back he used . . . the TENS machine, [which] means he can drive home’* (P1).

*'I did let my sister have, yeah, she had a go at [the stair lift], because she’s got a problem with her back or knee’* (P8).

Another example of the potential of how medical devices were used to bring people together is the way they became part of the interaction between the grandchildren and their grandparents:

*'Yeah, well, yeah, yeah, he just says you know, 'Granddad, what are you doing? Oh you’re doing your injection [using the blood glucose monitor] are you? Let me watch.’* (P2).

*'[my granddaughter] sits at the side of me when I’m having one [a nebuliser]’* (P10).

Other respondents used the device with their grandchildren as part of play:

*'And he said, “Can I have a go at this [stair lift] Nanny?” I said, “Yes, sit on it,” so he had a go on it. [He said], “This is good, isn’t it? It doesn’t go very fast.” I said, “No, I’m not meant to go very fast!”* (P8).

*'My grandchildren love it because they have a go on it when they come out and do their blood pressure. They have a little book that I keep their blood pressure in when it’s done’* (P12).

This theme has described how the medical devices in this study had an effect on the social lives of users and partners. These effects can be seen to be both negative in that they act as a barrier between people or positive in the way they can bring people together.

## Discussion

In the first theme of 'self-esteem’ there was a real sense of powerlessness when participants were talking about their illness and that there was no real alternative to having these medical devices in their lives. Being diagnosed with a chronic illness can undermine one’s sense of personal control and lead to tension and psychological distress including decreased levels of self-esteem [18,19]. Part of the adjustment process to a threatening event (e.g. chronic illness) is control or 'mastery’ over the event in order to manage the condition and express a sense of personal control [20,21]. This can be seen in the present study by participants using the device to monitor their condition and using the results to modify behaviour. Another expression of mastery can be achieved by assuming control over related aspects of an illness such as treatment and becoming an expert in how to use the device itself.

This theme also described examples of where the medical device facilitated participants' ability to derogate others and point out that other people with similar conditions and devices were doing less well or using their devices incorrectly. This is consistent with the literature in that when faced with a threat to self-esteem, individuals can often make downward self-enhancing comparisons that increase subjective well-being by comparing themselves with less fortunate others [22]. Similarly, individuals may also derogate or devalue other persons in a bid for self-enhancement [23].

Device use also impacted on the lives of device users’ partners and wider social circle as described in the second theme 'the social device’. The literature has tended to focus on that of the role of 'family caregiver’, an individual who lives with or close to the ill person who provides unpaid assistance with social and medical care [24]. Studies that have looked at spousal carers of individuals suffering from chronic diseases have noted the impact of role change as the role of husband/wife is eroded and is absorbed in to that of carer [25]. Little evidence was found to support this suggestion in this study as the users required very little in the way of assistance and the role of husband/wife remained intact. The reason for this is unclear but could be the result of the equipment acting to supplement informal care and promote independence [26,27]. This meant the relationship between the spouse and the device was different to that of the relationship between the device and the user. In this study the spouse and user are required to share the same physical environment and the resultant consequences of noise and aesthetics with the spouse not directly benefiting from device use (any benefits from the device for the spouse would be deemed as being indirect). The fact that some of these devices were used in the shared bedroom meant that sleep was disturbed or sleeping arrangements had to be altered (changing sides of the bed, sleeping in another room). There were also other emotional consequences of device use on partners. While one husband reported a certain amount of embarrassment when out with his wife while she was using her device, another user reported the very act of using the device in front of her husband caused the husband to become annoyed. While this may have been an expression of concern for his wife’s health it could also be that it had a detrimental effect on the way he thought about himself. Steele, Spencer and Aronson [28] proposed that situational cues that identify a person as belonging to a devalued, low status social group lead to social identity threat. In this case conspicuous medical device use might have caused discomfort because it reminded him of his own age and mortality. The influence on spousal identity has also been discussed in relation to assistive devices used by people suffering a stroke which reported similar findings [29].

The effect on others was not entirely negative. One example of the positive effects on others was the benefit they received from the shared use of medical devices with regards to symptom relief or physical function. Also, the use of devices in some cases was not hidden away from grandchildren but actually became a part of the social interaction with the grandchildren 'helping’ with the equipment or 'using’ it as part of supervised play. While much has been written around how families experience chronic illness (for a review of the literature see [30]) little has specifically addressed the effect of medical devices on the relationship between grandparent and grandchild. The ways that devices were being used in play may be an example of normalization strategies used to decrease disruption and maintain family processes [31].

In general, participants expressed positive feelings about their devices and the impact that the device had on their lives, and the users themselves expressed an overall feeling of satisfaction and were often not able to think of ways to improve the device. A possible contributing factor to this might be found in the literature that has identified a link between age and satisfaction. Analysis of patient satisfaction surveys in the UK identifies age, as opposed to other socio-demographic factors, as having the strongest influence on satisfaction levels. Older people are consistently more likely to express greater levels of satisfaction with regards to health care than younger people [32]. This may in part be due to older people’s greater experience of healthcare and its potential shortcomings resulting in decreased expectations and a resultant increase in their satisfaction [33].

There is no doubt that the emotional content of design in everyday products is becoming increasingly important but it is proposed that the reaction to meaning and emotional response that is triggered by a particular product varies between generations, cultures, social groups and nationalities [34]. This implies that devices could be specifically designed for older people in order to prompt a positive emotional response.

### Limitations

The use of opportunity and snowball sampling might have limited the diversity of participants and consequently the generalizability of the findings. Anecdotally, many of the participants in this study reported that they had replied to the adverts out of a sense of 'gratitude’ and wanting to give something back which might have biased the interviews. Gratitude bias can be seen, particularly in public funded service such as the National Health Service, when participants are reluctant to criticise and have a tendency to gloss over any negative concerns they have about services and treatments because they feel they should be grateful with what they receive [35]. Whether this biased the results of this study is hard to determine however, as on the whole there did seem to be a great deal of praise for the treatment they received and the healthcare staff they encountered.

While interviewing the user and partner together allowed them to prompt and remind each other of instances and issues during the course of the interview it may also have resulted in a less open and frank discussion. For example it might be very hard for a next of kin to express negative experiences and emotions of having the technology in the home in front of the user, who may be medically dependent on the device. In general the older people in this study seemed less willing to talk about their feelings towards their medical devices than was expected. There also seemed a difficulty in focusing the talk on the device itself as opposed to the medical condition which demonstrates the inextricable links between the condition and device, and vice versa.

While the use of thematic analysis allowed for a rich description across the data set, the nuances contained in individual accounts tend to be lost, thus limiting complexity and the reporting of individual aspects of the situation from within the data. In this case thematic analysis was used to provide a broad description of the interview content as opposed to making any high order theoretical claims.

## Conclusions

This study has described some of the challenges faced by this particular group of older people in relation to medical device use. The challenges include the social consequences and effect on self-esteem that are a result of these devices entering their lives. This change in situation from non-user to user can also have implications on the home in which they live and the people they share their life with.

The idea that many of the devices in this study are regarded as 'home use’ and have been designed to slot into people’s lives is not generally supported by these older people. In fact the opposite seems to be the case with reports of adjustments and alterations having to be made to people’s lives to accommodate devices into the home.

Simply addressing the clinical and safety requirements of users in relation to the design of these medical devices does not guarantee user satisfaction, and the importance of considering the context in relation to the device/user interaction is gaining prominence. Further studies examining how medical devices can better fit in to the home and how other family members, not just the users, feel about sharing their lives with such devices are required. Longitudinal studies designed to capture the experience of change from non-user to user of medical devices may be particularly useful. This may encourage not only a more sympathetic design that both reduces the disruption to older people’s lives and facilitate greater satisfaction but also enable healthcare professionals to better prepare older people for the integration of medical devices into their lives and homes.

## Competing interests

The authors declare that they have no competing interests.

## Authors’ contributions

RT conceived of, designed and carried out the study. RT also analysed and interpreted the data and contributed to the writing of the manuscript. JM and SS conceived of and designed the study and contributed to the writing of the manuscript. All authors read, critically assessed and approved the final manuscript.

## Pre-publication history

The pre-publication history for this paper can be accessed here:

http://www.biomedcentral.com/1472-6963/13/467/prepub
